# The Zebrafish Disease and Drug Screening Model: A Strong Ally Against Covid-19

**DOI:** 10.3389/fphar.2020.00680

**Published:** 2020-05-01

**Authors:** Jorge Galindo-Villegas

**Affiliations:** Genomics Group, Faculty of Biosciences and Aquaculture, Nord University, Bodø, Norway

**Keywords:** SASRS-CoV-2, COVID-19, drug screening, animal models, zebrafish, pandemic

## Opinion

By April 21, 2020, the official number of Covid-19 cases comprised of 2,397,217 confirmed cases and 162,956 fatalities worldwide (W. H. O., 2020). While waiting for a viable vaccine, the disease severity continues to devastate the world population following a critical exponential trend. Open perspectives on neutralizing the right antigenic structural protein of severe acute respiratory syndrome coronavirus 2 (SARS-CoV-2) are broad. Several protein targets exist, including the nucleocapsid, spike, envelope, membrane, and nine putative accessory factors of the virus. However, so far, leading efforts in producing a vaccine mainly focus on the principal target of the humoral immune response, the glycosylated SARS-CoV-2 spike trimer, that mediates cell entry and membrane fusion. Although unusual, based on past experiences, highly respected researchers now believe this approach may lead to the opposite intended effects and result in rapid initiation of a macrophage activation syndrome (MAS) (Iwasaki and Yang, 2020). In the worst scenario, MAS ultimately switches macrophages from a wound-healing beneficial behavior to a proinflammatory phenotype, exacerbating the host. Patients with MAS overproduce proinflammatory molecules like transferrin, IL-6, IL,8, and MCP1, and lose the critical tissue-repair cytokine TGFb. Together, this would generate a worsening of the disease and, thus, be an immediate threat to life.

Just recently, efforts on an alternative promissory approach was approved. The principle is simple. Recycling established pharmacological drugs and clinical molecules that might perturb the interactome of SARS-CoV-2 patients diagnosed with Covid-19 could provide relief on their pathological status. So far, a French research team has provided new but questionable (https://blogs.sciencemag.org/pipeline/archives/2020/04/11/the-latest-hydroxychloroquine-data-as-of-april-11) conclusions by following an open-label non-randomized clinical trial using a few individuals infected with Covid-19 and hydroxychloroquine and azithromycin as a treatment (Gautret et al., 2020). A second-team tested the protein-protein interactions between human embryonic kidney cells (HEK-293T) and SARS-CoV-2 infection, uncovering several different drugs and compounds of potential clinical value (Gordon et al., 2020).

Furthermore, one more research group tested the FDA-approved drug Ivermectin on the SASRS-CoV-2 replication inhibition using the African green monkey kidney VERO/hSLAM cells (Caly et al., 2020). However, at this stage, the three approaches present severe constraints. Testing a broad panel of chemical drugs directly on volunteer patients includes critical issues surrounding the patient’s physiological life, treating risk, genetic differences, and the danger of strong ethical considerations. With this approach, while recommendations exist to screen the compounds at early stages by using *in vitro* cell cultures, and HEK-293T or VERO/hSLAM kidney cells are permissive to SARS-CoV-2 infection, they do not represent in any way the primary physiological site of infection and fully overpass the knowledge that a full live organism can provide *in vivo*.

Animal models of human diseases have proven effective in the screening of pharmacological drugs and in further determining the specific therapeutic targets in humans. Therefore, a collection of reliable animal models that replicates the Covid-19 clinical symptoms is urgently needed in order to decipher the transmission route and pathobiology of the disease. Among the available model candidates, a wide selection exists with murine models on top of the list (Callaway, 2020). However, a handy vertebrate, the zebrafish, emerges as an outstanding and specific poised model for the screening of novel therapeutics and chemical modifiers of biological processes *in vivo*. The zebrafish is an extremely versatile organism that, following proper automated settings, can be visually inspected using transmitted light or fluorescent imaging. At the same time, the signaling pathways, chemical interactions, and physical forces that regulate the development and progress of the host-virus interactions on mucosal tissues are amenable to analysis with exquisite detail. Indeed, the process of chemical throughput screening using the full organism is simple. The early developmental stages behave in a well-plate similarly to the cells used in must laboratories practicing *in vitro* testing approaches with pharmacological drugs. As a matter of support, it is worth noting that no matter the physiological differences, 84% of human genes associated with the specific disease have a counterpart in zebrafish, and the crosstalk of viral glycoproteins at the fish nasal neuro-immune edge are understood (Sepahi et al., 2019). Therefore, reliable projections suggest that employing zebrafish for analyses may provide faster, more accurate, reliable, and holistic responses compared with single-cell entities.

Several human viruses colonize zebrafish, such as Sindbis, chikungunya, and influenza A, as well as a variety of airborne bacteria and fungi (Gomes and Mostowy, 2020). A variety of mycobacteria affecting humans have been studied using this model on a level 3 biosecurity facility. Tuberculosis pathogenesis is a sound example, and the infection is established in zebrafish larvae using *Mycobacterium marinum* (a close relative of *Mycobacterium tuberculosis*), then screened *in vivo* by real-time fluorescent microscopy and automated plate fluorometry (Takaki et al., 2013). Infection clearance was achieved following an *in vivo* screening of large chemical libraries on 96-well microplates, with only 0.1 µmol of drug per fish. Among the drugs recently proposed for use against Covid-19 (Gordon et al., 2020), metformin (Met) has been suggested. Potentially beneficial effects of Met on cellular metabolism, immune function, and gene transcription involved in innate host responses to *Mycobacterium tuberculosis* pathogenesis has been successfully screened, linking the core of radially arranged microtubules at the axoneme of variated human respiratory diseases with the zebrafish.

No matter how severe the impact of Covid-19 is, the association of the incredible medical power in several profoundly impacted nations possessing zebrafish facilities working together with world-top scientists (like those at the Boston Children Hospital in the USA, Karlsruhe Institut für Technologie in Germany, Hospital for Sick Children in Canada, Regenerative Medicine Institute in Australia, Western General Hospital in the UK, or Hospital Universitario Virgen de la Arrixaca in Spain) with cutting-edge technology and full capacities in the use, the application of the above-described zebrafish techniques could provide novel results soon.

We face a huge public health emergency (Song and Karako, 2020), thus, the sole intention of this perspective is to urgently inspire the medical community to explore the compound effect and associated molecular mechanisms of the proposed pharmacological drugs on this alternative approach committed toward fighting the pandemic.

## Author Contributions

JG-V designed, drafted, and submitted the full document.

## Conflict of Interest

The author declares that the research was conducted in the absence of any commercial or financial relationships that could be construed as a potential conflict of interest.
